# Progress in the photodynamic therapy treatment of Leishmaniasis

**DOI:** 10.1590/1414-431X2021e11570

**Published:** 2021-10-29

**Authors:** D.G. Vital-Fujii, M.S. Baptista

**Affiliations:** 1 Departamento de Bioquímica, Instituto de Química, Universidade de São Paulo, São Paulo, SP, Brasil

**Keywords:** Photodynamic therapy, Photosensitizer, Cutaneous leishmaniasis

## Abstract

Leishmaniasis is a serious and endemic infectious disease that has been reported in more than 90 countries and territories. The classical treatment presents a series of problems ranging from difficulty in administration, development of resistance, and a series of side effects. Photodynamic therapy (PDT) has already shown great potential for use as a treatment for leishmaniasis that is effective and non-invasive, with very minor side effects. PDT can also be inexpensive and easy to administer. In this review, we will report the most recent developments in the field, starting with the chemical diversity of photosensitizers, highlighting important mechanistic aspects, and noting information that may assist in designing and developing new and promising photosensitizer molecules.

## Introduction

Leishmaniasis is a group of neglected diseases caused by parasites of the genus *Leishmania*. More than 20 species are found worldwide, while the main species found in Brazil are *Leishmania (Viannia) braziliensis*, *L. (V.) guyanensis*, *L. (V.) lainsoni*, *L. (V) naiffi*, *L. (V.) lindenberg*, *L. (V.) shawi*, *L. (Leishmania) amazonensis*, and *L. (L.) infantum chagasi*. These parasites belong to the Trypanosomatidae family and have two forms: the promastigote (flagellated form found in the vector's digestive tract) and the amastigote (intracellular spherical non-flagellated form lodged in macrophages) (1-4).

Transmission occurs through the bites of female sandflies, and the reservoirs of the parasites are humans, wild rodents, marsupials, and domestic dogs. There are three main forms of the disease: cutaneous, visceral or kala-azar, and mucocutaneous (1-4).

It is estimated that one billion people live in endemic areas, and more than 80 economically vulnerable countries are affected by visceral leishmaniasis (VL). With one million new cases of cutaneous leishmaniasis (CL) annually, the most affected countries are Brazil, India, Ethiopia, Kenya, South Sudan, Somalia, and Sudan (1,2,4).

The main symptoms of CL include single or multiple ulcerous or nodular lesions on the skin, which can become chronic or heal spontaneously six months after the sandfly bite. The condition is not fatal but it can be disabling and leave permanent scars (1,2).

In VL or kala-azar, a systemic parasite infection occurs, with symptoms such as enlarged organs, especially the liver and spleen, as well as prolonged fever, weight loss, and anemia. VL can also affect the lymph nodes and bone marrow, leading to death when left untreated. A major problem with this form is the risk of co-infection, for example, with HIV. Such co-infections have already been reported in 35 endemic countries worldwide (1,2).

Despite the toxicity, high cost, and difficult administration, treatment is still carried out with pentavalent antimonials, amphotericin B, pentamidines, and miltefosine. It is therefore necessary to find effective, safe, low-cost, and short-term treatment, of which photodynamic therapy (PDT) is a promising candidate (1,2,4).

PDT is a technique used in the treatment of several diseases. It uses electronic excitation of a photosensitive compound (photosensitizer) to produce a variety of reactive oxidant species (ROS), including excited states, free radicals, and strong oxidants (5-8). It is one of the most promising strategies for local anti-microbial therapy and killing drug-resistant microorganisms (9). Compared with conventional therapies, PDT is a low-cost, minimally invasive technique with minor side effects (9). Since CL is a local infection, PDT has been studied by several researchers and clinical groups as a treatment alternative (10,11).

**Figure 1:**
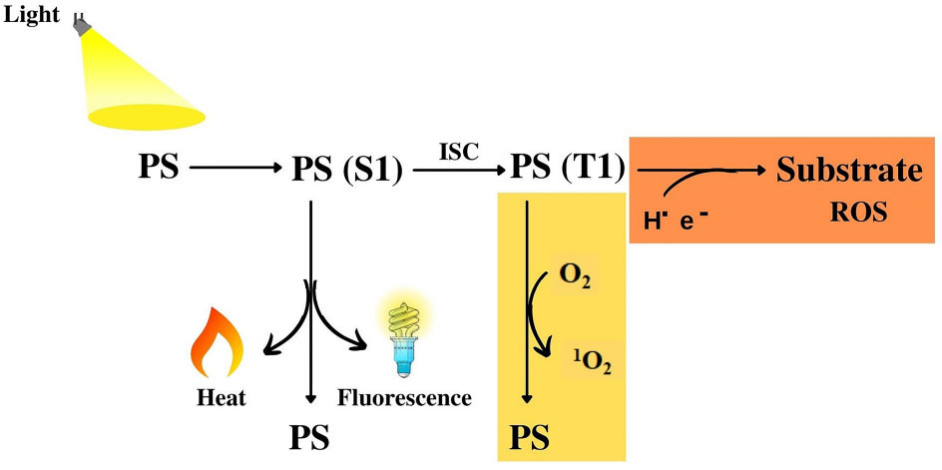
Photosensitization process. PS: ground state photosensitizer; PS (S1): singlet state photosensitizer; PS (T1): triplet state photosensitizer; ISC: intersystem crossing; orange box: type I reaction; yellow box: type II reaction.

PDT has three essential components - the photosensitizer, the light, and the molecular oxygen. The photosensitization process (Figure 1) starts with the absorption of light by the photosensitizer, raising it to the singlet excited state (S1), which can return to the ground state by losing heat or emitting light (fluorescence). S1 state may suffer changes in the electron spin (intersystem crossing), forming an excited triplet state (T1) with a longer lifetime. T1 has time to react and diffuse, either by transferring energy to molecular oxygen and forming singlet oxygen (Type II reaction) or by transferring electrons (or hydrogen) to a substrate (Type I reaction). The electron transfer reaction forms radicals and produces ROS after a subsequent reaction with oxygen, such as the superoxide radical anion, hydrogen peroxide, and the hydroxyl radical (12,13).

Photosensitized oxidations, which are reactions triggered by the interaction of light with photosensitizer (PS) molecules, act by inducing damage in cytoplasmic or organelle membranes. These are key to modulating the mechanism and overall efficiency of regulated cell death (14). Type I reactions consist of the direct oxidation of biological targets (direct-contact reactions), while those of type II involve oxidation mediated by diffusing species (independent-contact reactions), mainly the singlet oxygen. In direct-contact reactions, the damage occurs at the exact point where the excited species are generated. In contrast, singlet oxygen or other diffusing species can carry oxidation potentials hundreds of nanometers or micrometers away from the point of light absorption (15). Nevertheless, the detailed molecular steps leading to biological injury remain largely uncharacterized, and the level of precision in the spatial damage caused by photosensitized oxidation reactions remains unclear.

For a PS to fully compromise membrane function, it needs to engage in electron transfer reactions with either the lipid double bond or the lipid hydroperoxide. This process forms peroxyl and alkoxyl radicals within the membranes that undergo Beta-scission and generate lipid-truncated aldehydes, which cause membrane leakage (16). Therefore, cellular damage occurs precisely at the PS locus. This highlights the importance of finding molecule-specific oxidation-induced photodamage. Since the efficiency of membrane leakage results from an electron transfer reaction that usually causes photobleaching, PS regeneration should be exploited as an effective tool to develop improved PDT photosensitizers (16,17).

**Figure 2:**
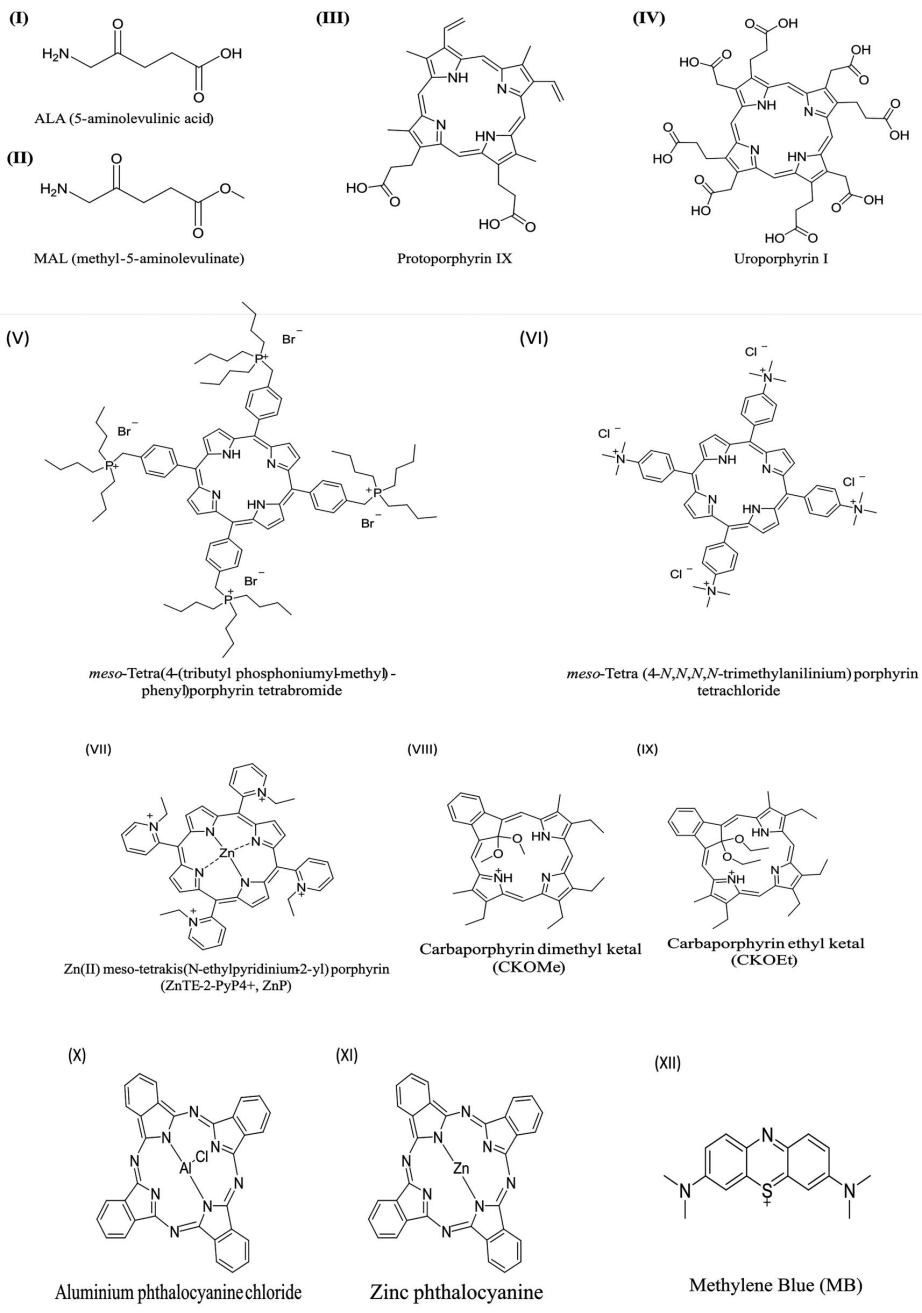
Structure of presented photosensitizers. **I**-**IV**, delta-aminolevulinic acid (ALA) and ALA derivatives; **V**-**IX**, porphyrins and derivatives; **X** and **XI**, phthalocyanines; **XII**, phenothiazinium salt.

Many series of PSs that are candidates for PDT are being tested as potential treatments for CL. Understanding how each class works will facilitate the design and development of the PS and improve PDT results. A growing body of literature has examined the use of various PSs in treating leishmaniasis, other infectious diseases (5,18-20), and cancer (21-23). In this review, we aim to describe and discuss the main achievements and challenges of using PDT to treat CL by examining recent results and a contemporary view of the mechanisms of the major series of photosensitizers, including phenothiazinium salts, delta-aminolevulinic acid (ALA) and ALA derivatives, phthalocyanine, porphyrin, and phenothiazinium (Figure 2).

## ALA-PDT

*Leishmania* species are known to use tetrapyrroles to promote the growth of promastigotes and to transform amastigotes into promastigote forms. Tetrapyrroles are not acquired from heme biosynthesis since *Leishmania* spp. entirely lack or are deficient in seven of the eight enzymes in the heme biosynthetic pathway (24,25).

A study involving *Leishmania (L.) amazonensis* showed that the parasites become sensitive to UV irradiation due to the presence of uroporphyrinogen I (URO), which is a by-product of hydroxymethylbilane (heme group synthesis) inside the parasitic cells that causes a loss of motility in *Leishmania* parasites (25). These findings showed the potential of porphyrins as photosensitizers in *Leishmania* PDT.

The first step in heme biosynthesis is the formation of ALA, which is caused by the condensation of glycine and succinyl-CoA in a process catalyzed by the enzyme ALA synthase (ALAS) (26-28). The latter is a mitochondrial enzyme that undergoes negative feedback from heme as it can induce or inhibit the enzyme once present, interrupting the production of ALA (28,29).

Although ALA is not a photosensitizer, it is the first precursor in the biosynthesis of the heme group and the universal precursor of tetrapyrroles like porphyrins (26,30,31). It became the target of PDT studies after it was discovered to be converted into protoporphyrin IX (PpIX) in heme synthesis, accumulating in some cells and acting as a photosensitizer (30,32). The synthesis of PpIX is determined by the amount of ALA produced, which is regulated in turn by the concentration of the free heme group. However, this feedback mechanism can be circumvented by the administration of exogenous ALA that induces the production and accumulation of PpIX or by certain mutations in the ALAS, which also cause PpIX accumulation (30,33).

Akilov et al. (34) performed a well-documented study on the action of PDT using ALA (termed ALA-PDT) in CL and a cellular assay. In the *in vivo* studies, the authors observed the formation of inflammation and necrosis, indicating damage to vascularized areas. Furthermore, ALA-PDT was found to decrease the parasitic burden 24-fold in the ear lesion. However, in the *in vitro* studies, the results showed that the amount of PpIX that *L. (L.) major* was able to obtain from the host cell was not sufficient to produce a photodynamic action (500-fold less than the amount needed to kill metacyclic forms). The results suggest that the success of the *in vivo* ALA-PDT is due to indirect action such as immune modulation since the PDT was not able to kill parasites.

As mentioned above, *Leishmania* spp. needs a supplementary source of tetrapyrroles (24,25), meaning that producing PpIX from ALA may not be possible (31).

In agreement with the results previously found by Akilov et al. (34), another study was performed using ALA and the derivative MAL (methyl-5-aminolevulinate) (35). Although these exhibited an internalization of the PS, no photodamage was observed in the promastigote forms, confirming the inability of *Leishmania* spp. in converting ALA to PpIX. However, exogenous PpIX was able to provoke phototoxicity in parasites.

In contrast to results found for the promastigotes, infected cells produced PpIX but not in sufficient amounts to promote damage on intracellular amastigotes (35), consistent with previous findings (34). Despite MAL having an extra alkyl chain in the structure, no difference was found in the internalization or photoactivity between the two compounds (35).

As previously seen, ALA-PDT may act through an immune modulation mechanism when applied directly to the lesion (34). This hypothesis was supported by some results that demonstrated a decrease in the parasitic load following an increase in interferon-gamma (INF-γ) levels after ALA-PDT of infected mouse paws (36). The increase in INF-γ levels is described as causing resistance to infection by *L. (L.) major* due to the development of protective immunity (37).

Silva et al. (38) studied macrophage modulation in mice infected by *L. (V.) braziliensis*, the action of ALA-PDT in the lesions, and whether ALA-PDT could alter heme oxygenase 1 (Hmox-1), an enzyme responsible for the persistence of infection and inflammatory response activation (39).

As seen in the previous study (36), the parasite burden decreased significantly compared to the untreated group, and Hmox-1 levels were not significantly altered, indicating no association of the enzyme with the persistence of the infection or any decrease in the parasite load (39).

However, a notable increase in iNOS (nitric oxide synthase) and iNOS/arginine ratio was observed. This could be associated with the ability of ALA-PDT to kill parasites since iNOS catalyzes the production of NO (nitric oxide), which is a leishmanicidal agent. Together with the earlier study, these findings show an association between ALA-PDT and the activation of macrophages that release leishmanicidal mediators. Taken together, these effects increase parasite mortality and decrease the rate of parasitism (36,39).

ALA is not a PS and cannot inhibit the parasite itself since it is a prodrug, and the parasite is incapable of converting ALA to the photoactive product, porphyrin. However, it is an excellent option for use with PDT directly on the lesion as it causes an alteration in the immune response and decreases parasite load.

Although ALA does not kill the parasite, the structure can be used to develop a new PS, exploiting the capacity of ALA to be converted into photosensitive porphyrins. Porphyrin can be used as a PS in PDT against *Leishmania* spp. successfully.

## Porphyrins

Porphyrins have been described as an excellent choice for PSs since their effectiveness against *Leishmania* has already been demonstrated during PDT studies (25,35). One PDT study used exogenous PpIX as a positive control and tested against *L. (L.) infantum* and *L. (V.) panamensis* (35). Although PpIX was only used as a control, it showed promising results against the promastigote forms of *L. (L.) infantum* and *L. (V.) panamensis*.

Before this, Bristow et al. (40) studied four types of cationic porphyrin PSs as potential photodynamic anti-*Leishmania* agents. The cationic PSs were chosen due to the negative character of the membrane of *Leishmania* spp. and were tested against the promastigote forms of *Leishmania (L.) major*. Macrophages and keratinocytes were used to simulate amastigote intracellular forms of *Leishmania* and the healthy tissue around the skin lesion, respectively (40). PSs were tested and showed very different results, which was thought to be due to the differences in the membrane charge of the three cell lines studied. As noted, the cell membrane of the *Leishmania* spp. has an anionic character (41).

Compounds 1 (phosphorous-centered cationic porphyrin) and 3 (nitrogen-centered cationic porphyrin) were photoactivated against the promastigote forms at a low concentration. However, compound 1 showed no activity while compound 3 inhibited macrophages with a similar LD_50_ for *L. (L.) major* promastigotes (40). Meanwhile, compound 1 was active against keratinocytes at a concentration equivalent to almost half that needed to kill promastigotes, but compound 3 reached inhibition at a concentration 10 times higher than the one established for promastigotes. Since compound 3 had the best results, a dose adjustment was considered so that it could be used to kill promastigote and amastigote forms without causing damage in uninfected tissue (40).

In 2018, Andrade et al. (42) studied the effect of zinc porphyrin (ZnP), a cationic porphyrin, to verify the effect of the charge and the zinc on membranes. ZnP was active in both tested concentrations of about 65 and 90%. Furthermore, an analysis of the parasitic cells after 24 h of PDT showed that cells were incapable of replicating. These results were successful because the PS concentration was low, the incubation time was only 10 min, and the irradiation time was short (42). This treatment exhibited high permeability in the parasitic membrane. The microscopic alterations included a shortening and rounding of the parasitic cells, shrinkage of the plasmatic membrane, and vacuolization. ZnP was less cytotoxic than compounds 1 and 3 described by Bristow et al. (40) Approximately 70% of human cells remained viable after ZnP PDT, while about 50% of cells showed viability after a cationic porphyrin assay (42).

Both studies (40,42) were important demonstrations of the effectiveness of porphyrins in PDT against *Leishmania*. It was possible to observe that cationic porphyrin is a promising PS for use against *Leishmania* spp. However, the former study (40) made it clear that the positive charge is not the only factor to be considered. Meanwhile, the presence of the metal in the second study (42) indicates that this strategy may be relevant in structure activity studies.

Carbaporphyrin ketals are porphyrin derivatives, called porphyrinoids, in which the pyrrole ring of porphyrin is replaced by a ketal-substituted indene ring. This class of compounds was tested *in vitro* and *in vivo* against *Leishmania (L.) amazonensis*, *L. (L.) infantum*, and *L. (V.) panamensis* (43). Such a study allows verification of whether the alteration in the activity is caused by the changes in compound structure.

The carbaporphyrin dimethyl ketal (CKOMe) and carbaporphyrin diethyl ketal (CKOEt) had high activity levels against axenic and intracellular amastigotes of studied *Leishmania*. When used in a liposomal formulation, PDT had a stronger effect against *L. (L.) amazonensis* for both compounds. However, similarly to the case of cationic porphyrins (40), carbaporphyrins were toxic to human cells, although the toxicity of CKOMe to PMH cells (peritoneal macrophages obtained from hamsters) decreased when administered in a liposomal formulation (43). Even though CKOEt is more hydrophobic than CKOMe, the latter showed better results, even when solubilized in DMSO or a liposome, showing that something other than hydrophobicity is important. The size of the molecule may be important here, as CKOEt has two ethyl chains that could cause a steric effect that would affect activity. The study also shows that altering the porphyrin backbone is useful for increasing activity against *Leishmania* species.

## Phthalocyanine

Phthalocyanine (Pc) is a synthetic dye that consists of four isoindole rings connected by four nitrogen atoms (44). Pc is an alternative to porphyrins since PSs do not absorb in the 400-600 nm range, having no phototoxicity to the skin. Unlike porphyrins, Pc has an absorption and fluorescence wavelengths in the range of 650-800 nm and high production of singlet oxygen. However, like porphyrins, Pc can form metal complexes that further increase the ability to produce singlet oxygen (44,45).

When exposed to aluminum phthalocyanine chloride (AlPhCl) and light, promastigote forms of *L. (L.) amazonensis* were rapidly killed, while axenic amastigotes underwent structural alterations. Both groups exhibited loss of fluorescence, indicating cell lysis. It is important to note that neither the light nor AlPhCl was toxic when administered alone, and both the dose of light and the Pc concentration used were low. In addition, AlPhCl reduced the number of macrophages and amastigote forms in an infected culture (46).

Escobar and colleagues (47) analyzed the photoactivity of aluminum chloride and zinc phthalocyanines (AlPc and ZnPc) against promastigote forms of *L. (L.) chagasi* and *L. (V.) panamensis*. AlPc had a dose-response activity and was more phototoxic to *L. (L.) chagasi* than *L. (V.) panamensis* (30- to 50-fold). AlPc also caused greater photosensitization than ZnPc in both parasitic species, and none of the PSs presented phototoxicity in the dark. The differences found between the two PSs could be related to the fact that ZnPc is very hydrophobic, which could make it difficult to enter the cell, unlike the amphiphilic AlPc (47).

To facilitate entry into infected cells, decrease the formation of dimers, improve photoactivity, and reduce phototoxic effects, Hernández et al. (48) reported the activity of ultradeformable liposomes containing chloroaluminum phthalocyanine (UDL-ClAlPc) and free ClAlPc against *L. (L.) chagasi* and *L. (V.) panamensis* promastigotes and amastigotes. UDL-ClAlPc was more phototoxic than free ClAlPc in both species and parasitic forms. As previously reported, ClAlPc showed no selectivity for the parasitic intracellular form compared to host cells (34,46). This result confirmed that the death of the parasite could occur due to a secondary mechanism.

The use of genetically modified *Leishmania* to produce and accumulate URO has been successfully described (25). Therefore, Dutta and colleagues decided to use this technique along with AlPhCl to increase parasitic photodynamic efficiency (49).

First of all, URO and AlPhCl alone showed a very different localization into cellular structures. As expected and described (25,47,50), the more hydrophobic AlPhCl showed a greater accumulation in structures such as the cell membrane, while the hydrophilic URO was more widespread in the cytoplasm (49). Promastigotes photosensitized with URO or AlPhCl alone showed a reduction in viability. Also, a total loss of cell viability was only possible after photosensitizing the cells with both PSs (URO and AlPhCl). This loss of viability was maintained after five days of culture, showing that the synergism technique may have photoinhibition applications. Notably, infected macrophages were not affected by this treatment, although all the parasitic intracellular burden was eliminated (49).

The fact that the photosensitizers were used together and the host cells were not affected is an important outcome (49), as previous studies (34,46,48) showed that PDT might have killed the parasites by a secondary route and not by directly acting on the parasite itself.

Ribeiro et al. (51) evaluated the effectiveness of the PDT technique when associating the topical use of AlClPC (liposomes containing chloroaluminium phthalocyanine) with the drug miltefosine, already used to treat CL (52-54). After 20 days of treatment with miltefosine and PDT with AlClPC, they noted a reduction in the diameter of the infected paw accompanied by a considerable decrease in parasitic culture.

A nanoemulsion containing ZnPc was tested to optimize PDT studies in the treatment of CL (55). First, free ZnPc was evaluated against promastigote forms of *L. (L.) infantum* and *L. (L.) amazonensis*; in contrast to previous findings (47), it was active in the presence and absence of light, although light caused greater inhibition of *L. (L.) infantum*. The results presented by nanoemulsion were even better after irradiation for *L. (L.) amazonensis* and *L. (L.) infantum*. As previously observed (46,48), free PS was toxic to macrophages with or without irradiation. Like free PS, the nanoemulsion also showed cytotoxicity but with a certain selectivity for parasites compared to host cells. A reduction in the parasitic burden of macrophages was also found (55).

Escobar et al. (56) built on these studies by analyzing the topical use of UDL-ClAlPc in BALB/c mice infected with *L. (V.) braziliensis* and *in vitro* activity in *L. (V.) braziliensis* promastigotes and amastigotes and mammalian cells. In the *in vitro* assay, the UDL-ClAlPc internalized both infected and uninfected cells, and was active in both parasitic and host cells after PDT, with no selectivity. UDL-ClAlPc also induced ROS generation in infected macrophages after PDT. Since no activity was found after treatment with UDL-ClAlPc and only low levels of ROS were produced without PDT, the authors suggested that ROS production after PDT may have been responsible for killing the parasites. Damage to the DNA was found with or without PDT, even in the empty UDL, but the mechanism behind the damage was not studied. The study did not find any effect in the BALB/c infected mice treated with topical UDL-ClAlPc and PDT, which was explained as possibly due to photobleaching or low penetration in the skin (56).

These results contrast with those found by Ribeiro et al. (51), who also used the PS within liposomes. The latter study (51) combined topical use of the liposome with miltefosine, which may have been responsible for reducing the lesions present in the animals. Another difference is in the type of formulation: the first study (56) used a lipid film while the second one (51) created a gel formulation. These differences may explain the effectiveness of the liposome on mouse lesions.

According to the excellent results obtained previously in a study of the association of miltefosine with PDT using AlClPC as PS (51), Ribeiro et al. (57) decided to evaluate the effectiveness of the association between AlClPC and PDT with the drug N-methyl glucamine (NMG) in mice infected with *L. (L.) amazonensis*. The standard recommendation of NMG for the CL treatment is 20 mg Sb^V.^kg^-1.^day^-1^ (58), so the researchers decided to test 20 mg Sb^V^/kg/day + PDT + AlPlPC (NMG20 + PDT) and 10 mg Sb^V.^kg^-1.^day^-1^ + PDT + AlPlPC (NMG10 + PDT). The latter concentration was used to verify whether the PDT can decrease the NMG dosage, which may diminish adverse effects.

The treatment with NMG20 + PDT decreased the diameter of the animal's paw in 60 days after the end of treatment, a decrease similar to the negative control. It also showed negative results for amastigotes and parasitic cultures after 20 days of treatment and 60 days after the end of treatment. Cell viability was reduced after 10 and 20 days of treatment, even for NMG10 + PDT, although only NMG20 + PDT could maintain this reduction at 60 days after the end of the treatment. The results of NMG10 + PDT were similar to those of NMG20 alone (standard treatment), suggesting that it could be used in the future to minimize the adverse effects caused by the drug alone. It is important to note that this decrease is related to PDT + AlPlPC, indicating that AlClPC may be of interest as a PS (57).

Phthalocyanines have proven to be an excellent PS for use in PDT against different species of *Leishmania*, although more in-depth studies should be carried out on the selectivity and toxicity in the host cells. However, studies involving liposomal formulations and the association with existing drugs for the disease have shown satisfactory results both in terms of reducing parasitic load and skin lesions, as well as reducing the usual concentration and thus achieving a possible improvement in adverse effects.

## Phenothiazinium salts

Phenothiazine derivatives have been extensively studied, showing promising results against bacterial activity. Changes in structure, including the addition of methyl, nitro, and primary amine groups, the positioning of these groups, and the hydrophobicity of the molecule can improve the effects (59-61). Studies on the differences in chemical structure have shown that spatial constraints and the geometry of the phenothiazine derivatives are also important, for example, in aggregation (62). In addition to antibacterial activity, phenothiazine derivatives have also been studied for the treatment with PDT of other diseases such as Kaposi's sarcoma, herpes, and diabetic foot (63-65).

Methylene blue (MB) is the most studied photosensitizer of the phenothiazine class against *Leishmania* species. MB is an interesting photosensitizer due to its high singlet oxygen quantum yield of around 0.5 (66) and its absorption band between 550 and 700 nm. MB is capable of forming dimers, although this characteristic depends on the concentration, the ionic strength, and the presence or not of charged interfaces. The absorption spectrum of monomers and dimers is different, with maximum absorption at 665 and 580 nm, respectively (67,68).

Song et al. (69) performed *in vitro* and *in vivo* studies to assess the effectiveness of PDT and MB as a PS for treatment of CL. Although MB was effective without irradiation, the half inhibitory concentration (IC_50_) value in the parasitic cells was reduced after irradiation in a dose-dependent manner. After the *in vitro* assay, one patient with three lesions caused by *Leishmania (L.) amazonensis* underwent treatment with a low concentration of pentavalent antimonial and PDT + MB, with one lesion treated with Sb^V^ alone. The results showed that although the lesions were reduced and cured with Sb^V^ treatment alone, PDT accelerated this process.

Peloi et al. (70) performed an *in vivo* PDT study using 10 nM of MB in a lotion or aqueous formulation in hamsters infected with *L. (L.) amazonensis*. MB alone could not decrease the animal's footpad size, while animals treated with MB and light showed a significant reduction in footpad size, with no differences between the formulations applied. Some 40% of the infected animals had ulcerated lesions, and after treatment with MB in lotion and water plus light, 40 and 50% of the lesions were cured, respectively, after 12 weeks of treatment. The analysis of the parasitic load in the spleen of infected and treated hamsters found no presence of the parasite regardless of the formulation used. However, parasites were found in lymph nodes of treated hamsters in a much lower percentage compared to untreated hamsters.

To verify how PDT and MB can interfere in the interaction between macrophages and *L. (V.) braziliensis*, *in vitro* studies were performed (71). The parasitic load was decreased both in the group that received only MB and in the group that received MB + light, within the first 24 h of infection. After 48 h of infection, there was a significant 38% reduction in the group that received MB + light compared to the other groups. A 33% reduction in infectivity was observed within 24 h of treatment with MB and 58% when using MB + light. The infection rate of parasite macrophages was 71% lower in the group that received MB + light than the control. Also, when compared to the group that received only MB, the infection rate was 48% lower in the group that received MB + light after 24 h of treatment.

The studies by Song et al. (69) and de Oliveira et al. (71) showed that, although MB has activity in parasitic cells, the use of PDT together with MB enhances its action, in addition to allowing the decrease in PS concentration. Furthermore, Song et al. (69) and Peloi et al. (70) found that the use of PDT and MB healed the wounds caused by *L. (L.) amazonensis*. Therefore, the use of PDT associated with the phenothiazine compound is effective in both *in vitro* and *in vivo* assays, helping to decrease the parasitic rate and infection rate, as well as reducing the concentration of drugs already used in current therapy.

Pinto et al. (72) carried out a study in which they evaluated the internalization and cell location of the MB along with cell viability and morphology after the application of PDT in the species *L. (L.) major* and *L. (V.) braziliensis*. The internalization results showed that PS does not accumulate in organelles but rather remains in the cytosol of the parasitic cell. The most intriguing results were demonstrated in the tests of cell viability and mitochondrial activity. Although mitochondrial activity was altered in the control groups, the trypan blue viability assay showed that MB is not toxic in any species without light exposure. Furthermore, after the application of PDT, viability was significantly reduced, showing that the variation in mitochondrial activity does not necessarily mean a change in cell viability.

These results are in contrast to those reported by Song et al. (69), who verified MB activity even in the dark after a mitochondria activity test using MTT. In their study, this mitochondrial activity did not always interfere with the cell viability itself since parasites were shown to be viable even with changes in the activity of the mitochondria. In addition to the above, PDT using MB as PS caused morphological changes in the promastigote forms of both species, suggesting interaction with the parasitic cell membrane (72).

Sbeghen et al. (73) conducted a study comparing the action of PDT + MB administered intradermally and topically in lesions caused by *L. (V.) braziliensis* on hamster footpads. The authors observed that the MB applied intradermally did not cure or decrease the lesion. However, topical treatment reduced and healed lesions in 30% of the animals after nine weeks. Similar to the findings of Peloi et al. (70), the parasitic burden observed in the lymph nodes and spleen was low for animals treated with topical MB. Also, the treatment restored the lesion area and decreased inflammation (73).

MB proved to be an interesting photosensitizer for the treatment of *Leishmania* along with the PDT technique. We found that the studies showed good results both *in vitro* and *in vivo*, leading to a decrease of parasite load, reduction of lesions in both animals and humans, as well as action on several *Leishmania* species.

## Concluding remarks

PDT has proven to be a useful technique for the treatment of CL since it has a low cost, is non-invasive, and has low toxicity compared to conventional therapies. Although some adjustments are necessary, the PSs studied so far have shown promising results. In this review, a range of PSs and several methodologies were explored. These studies are important to assist in the search for increasingly efficient PSs against parasitic forms.

**Figure 3:**
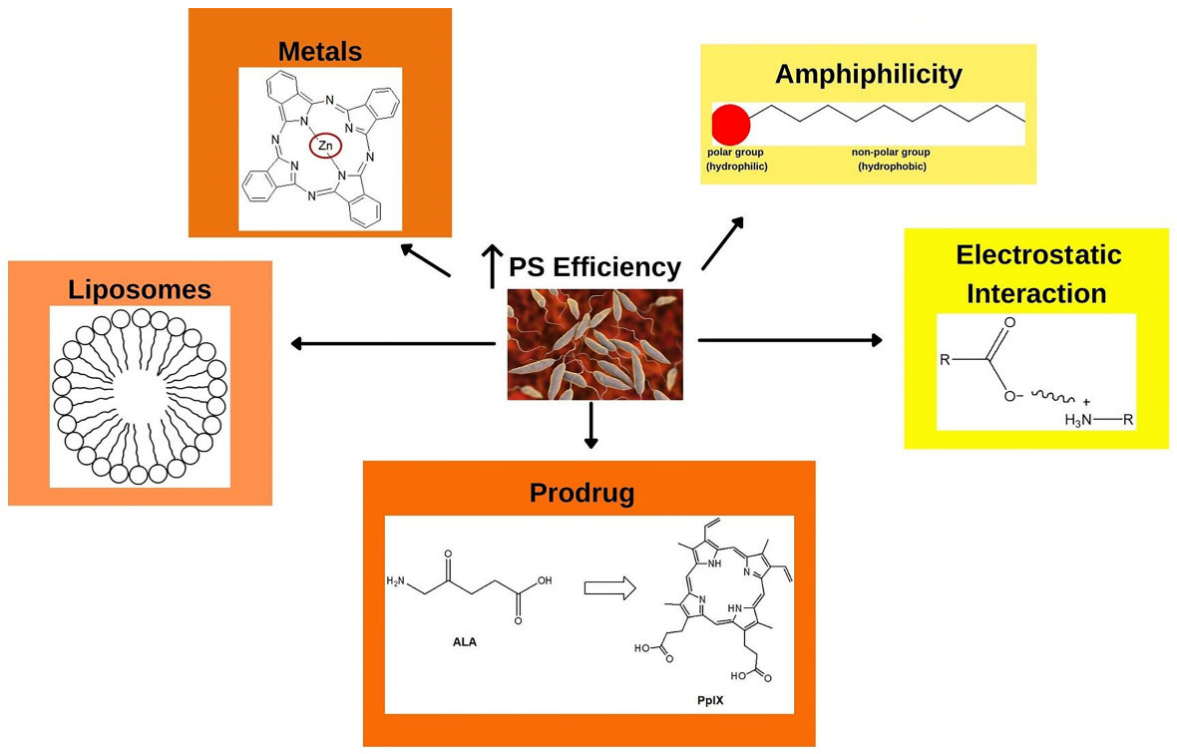
Key features to consider in increasing the effectiveness of photosensitizers (PS) against *Leishmania* spp.

We can highlight some of the aspects observed to be important in the development of a new PS, including the lipophilicity and amphiphilicity of compounds, the charges, the electrostatic interaction, and the presence or absence of metal (Figure 3). All of these factors interfered in some way during the studies.

As seen, porphyrins are active against parasitic forms, and a prodrug such as ALA can be used. Although it is not active on its own, ALA can be transformed into porphyrin (26,30,31). Furthermore, cationic porphyrins interacted better with parasitic cells since the parasite has a negative charge on its membrane surface, improving the electrostatic interaction of cationic compounds (40,41).

The presence of metal in the structure is another point to be considered, since an improvement in the activity of PSs has been observed. In addition, care should be taken when examining hydrophobicity, as highly hydrophobic molecules may not interact optimally. Like the amphiphilic molecules, they present better activity compared to hydrophobic molecules (42,47).

Finally, the use of techniques such as adding PSs to liposomes seems interesting. As we have seen in some studies, this strategy improves the efficiency of PSs and decreases toxicity (51,55,56).

Therefore, the study of PSs, especially in planning and development studies, should pay special attention to the efficiency in producing singlet oxygen while also adopting a molecular perspective, observing all the structural aspects important for the interaction between the PS and the target (17).
